# Supplementary material for: The herpesviral antagonist m152 reveals differential activation of STING‐dependent IRF and NF‐κB signaling and STING's dual role during MCMV infection
Source: EMBO J. 2019 Jan 29;38(5):e100983. doi: 10.15252/embj.2018100983 (PMC6396373; doi:10.15252/embj.2018100983)
Supplement: Supplementary file 1 — Appendix [file EMBJ-38-e100983-s001.pdf]

Appendix

Table of content

Appendix Supplementary Methods 2

Antibodies and reagents 2

siRNA knockdown combined with luciferase-based reporter assay 2

Appendix Figures 3

Appendix Figure S1: Luciferase-based screen to identify MCMV-encoded modulators of the cGAS-STING pathway 3

Appendix Figure S2: Protein expression of m152 and STING in stable cell lines used in this study (corresponding to Figure 1 and Figure 2) 4

Appendix Figure S3: Functionality of the generated STING mutants and quantification of CoIP experiments (appendix to Figure 4) 5

Appendix Figure S4: Luciferase-based reporter screen to identify a STING mutant with impaired IFN-inducing and preserved NF-κB inducing capacity (appendix to Figure 8) 6

Appendix Figure S5: siRNA-mediated knockdown of TBK1 does not affect STING-dependent NF-κB activation (corresponding to Figure 8) 7

Appendix Supplementary Methods

Antibodies and reagents

Rabbit anti-TBK1 (#3504, clone D1B4) was purchased from Cell Signaling Technology. Mouse antibodies against MCMV M45 (#HR-MCMV-13, clone M45.01) and MCMV M55 (#HR-MCMV-05, clone M55.01) were generated at the Center for Proteomics (CapRi), Faculty of Medicine, University of Rijeka. Phosphonoacetic acid, Cycloheximid and Actinomycin D were purchased from Sigma-Aldrich. siRNA against TBK1 (M-003788-02) and corresponding control siRNA (D-001810-10) were purchased from Dharmacon.

siRNA knockdown combined with luciferase-based reporter assay

293T cells were reverse transfected with siRNAs complexed with Lipofectamine. Per well of a 96-well plate, 0.05 µl of a 50 µM siRNA stock was combined with 9.95 µl OptiMEM. In parallel, 0.3 µl Lipofectamine 2000 were combined with 9.7 µl OptiMEM. Diluted Lipofecatmine was added to diluted siRNA and the mix was added to 293T cells. 48 hours post transfection, cells were transfected for a cGAS-STING luciferase reporter assay. For this, the siRNA-treated 293T cells were transiently transfected with 60 ng pIRESneo3 human cGAS-GFP (for stimulated conditions) or 60 ng pIRES2-GFP (for unstimulated conditions), 60 ng Cherry-STING, 1 ng p55-CIB , 10 ng pRL-TK, and 1.2 µl FuGENE HD (Promega) diluted to 10 µl total volume with OptiMEM. For NF-κB luciferase assays, cells were transfected as described above, but with 10 ng pNF-κB luciferase and 20 ng pRL-TK instead of 1 ng p55-CIB and 10 ng pRL-TK. 20 hours post transfection, cells were lysed and a dual-luciferase assay was performed.

Appendix Figures

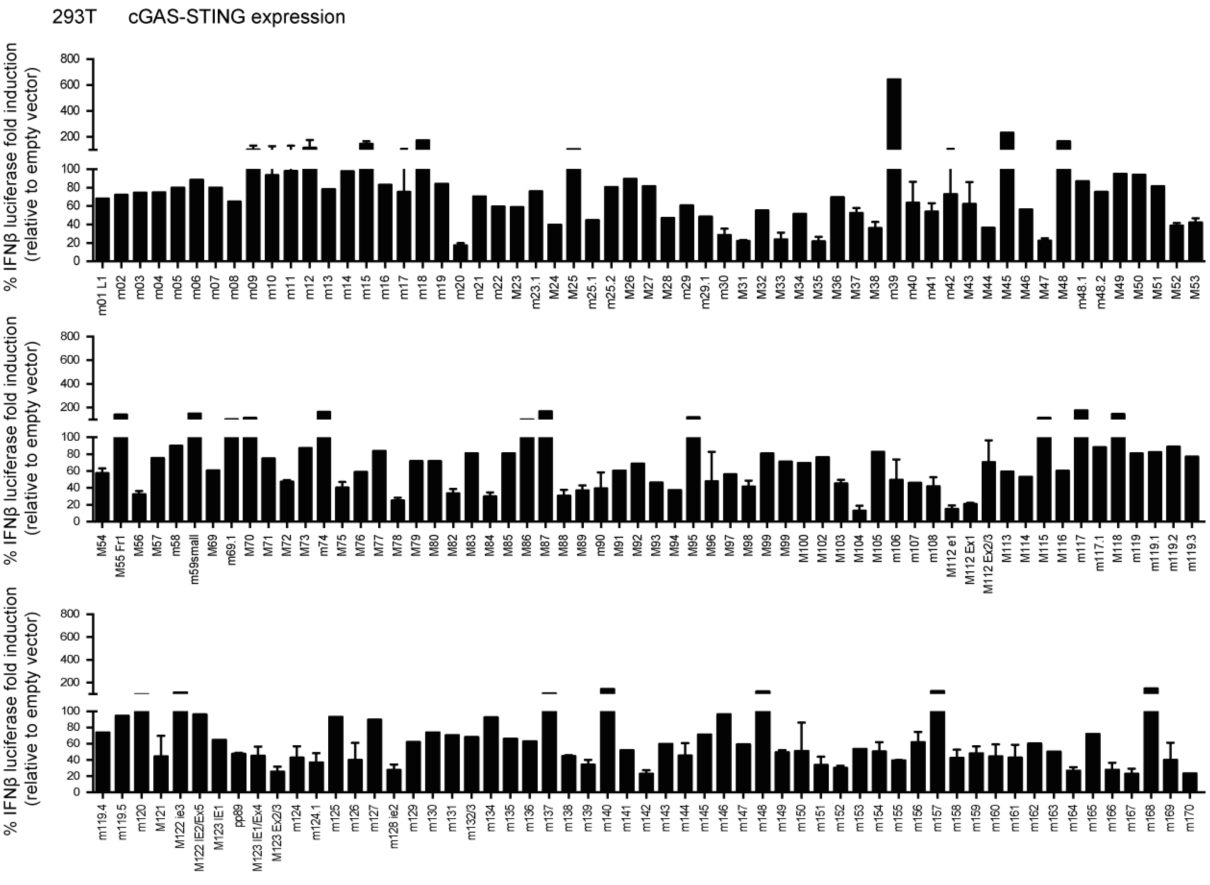

Appendix Figure S1: Luciferase-based screen to identify MCMV-encoded modulators of the cGAS-STING pathway

293T cells were co-transfected with expression plasmids for Cherry-STING, the murine IFNβ-luciferase reporter (IFNβ-Luc), a Renilla luciferase normalization control (pRL-TK) and the indicated MCMV plasmids. Empty vector instead of an MCMV plasmid was used for normalization. Cells were additionally co-transfected with expression plasmids for cGAS-GFP (stimulated) or IRES-GFP (unstimulated). 20 hours post transfection, cells were lysed and a dual-luciferase assay was performed. Data set is combined from one to four independent experiments and represented as mean ± SD.

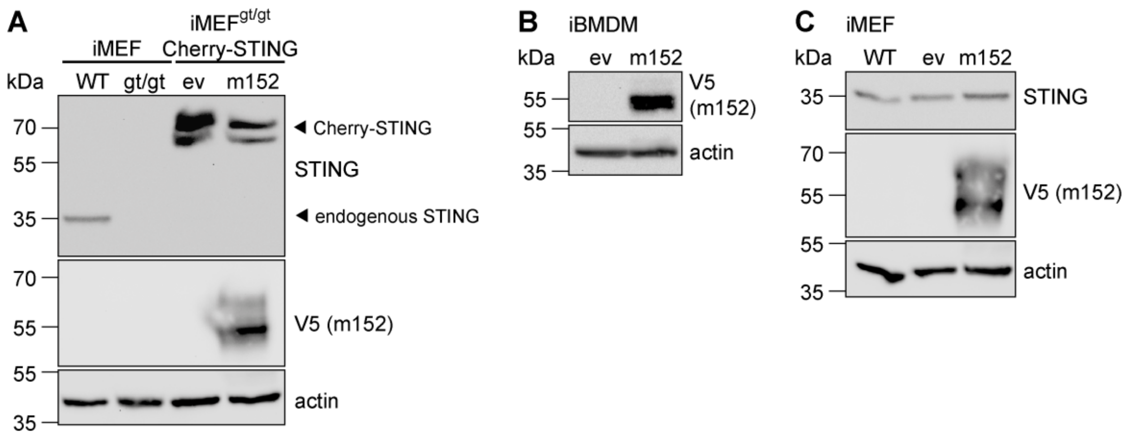

Appendix Figure S2: Protein expression of m152 and STING in stable cell lines used in this study (corresponding to Figure 1 and Figure 2)

(A) Cell lysates from the following cell lines were subjected to immunoblotting with antibodies specific for STING, V5, and actin: iMEF WT (lane 1), iMEF$^{gt/gt}$ (lane 2), iMEF$^{gt/gt}$ stably expressing Cherry-STING and ev (lane 3) and iMEF$^{gt/gt}$ stably expressing Cherry-STING and V5-tagged m152 (lane 4).

(B) Cell lysates from iBMDM stably expressing ev or m152-V5 were analyzed by immunoblotting with V5- and actin-specific antibodies.

(C) Cell lysates from the following cell lines were analyzed by immunoblotting with antibodies specific for STING, V5, and actin: iMEF WT (lane 1), iMEF stably expressing ev (lane 2) or m152-V5 (lane 3).

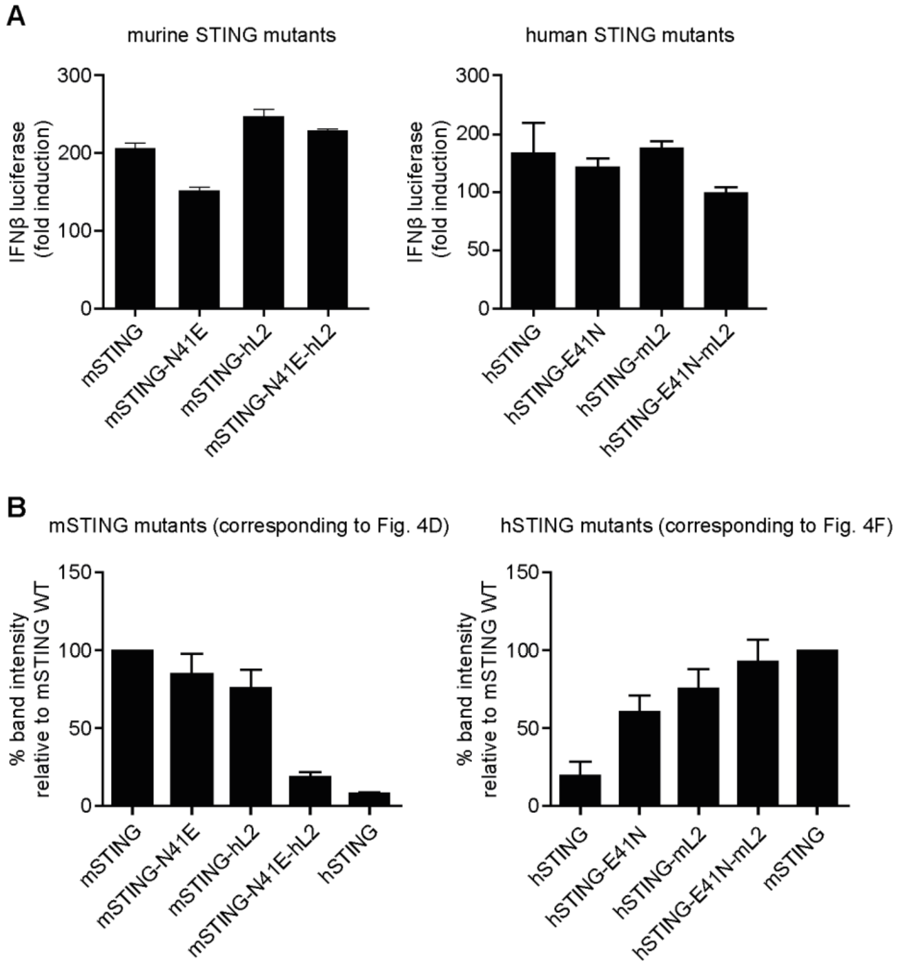

Appendix Figure S3: Functionality of the generated STING mutants and quantification of CoIP experiments (appendix to Figure 4)

(A) 293T cells were co-transfected with expression plasmids for cGAS-GFP (stimulated) or IRES-GFP (unstimulated) together with the murine IFNβ-luciferase reporter (IFNβ-Luc), a Renilla luciferase normalization control (pRL-TK), V5-tagged LacZ, and the indicated expression plasmids for the murine or human STING mutants or the respective wild-type. 20 hours post transfection, cells were lysed and a dual-luciferase assay was performed.

(B) The three individual co-immunoprecipitation experiments with loop mutants of murine and human STING (corresponding to Fig. 4D and Fig. 4F) were quantified using the Intas LabImage 1D software (version 4.1, build #3223). Band intensities were set relative to the band intensity obtained by the co-immunoprecipitation of MCMV m152 and mSTING WT. Data are shown as mean ± SD.

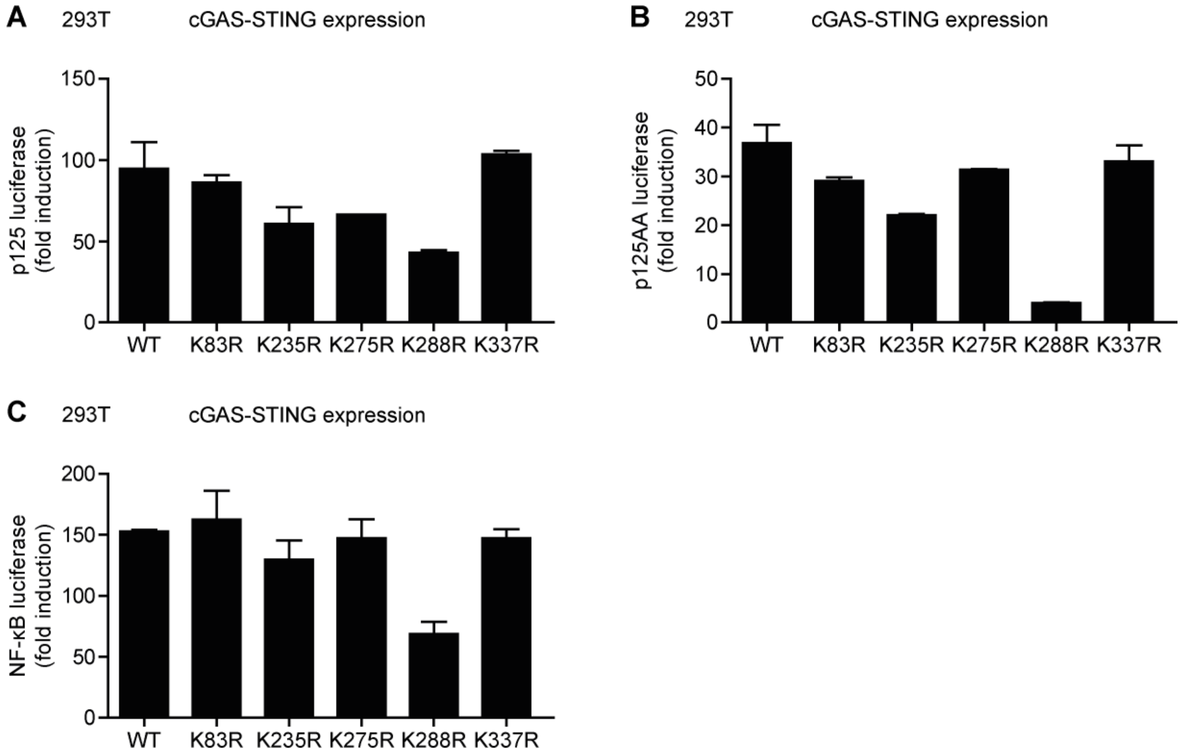

Appendix Figure S4: Luciferase-based reporter screen to identify a STING mutant with impaired IFN-inducing and preserved NF-κB inducing capacity (appendix to Figure 8)

(A-C) 293T cells were co-transfected with expression plasmids for cGAS-GFP (stimulated) or IRES-GFP (unstimulated), together with pRL-TK, WT murine STING or the indicated murine STING mutants and either the p125 (A), p125AA (B), or the pNF-κB luciferase reporter (C). 20 hours post transfection, cells were lysed and luciferase activity was measured. Data is representative from two independent experiments and shown as mean ± SD.

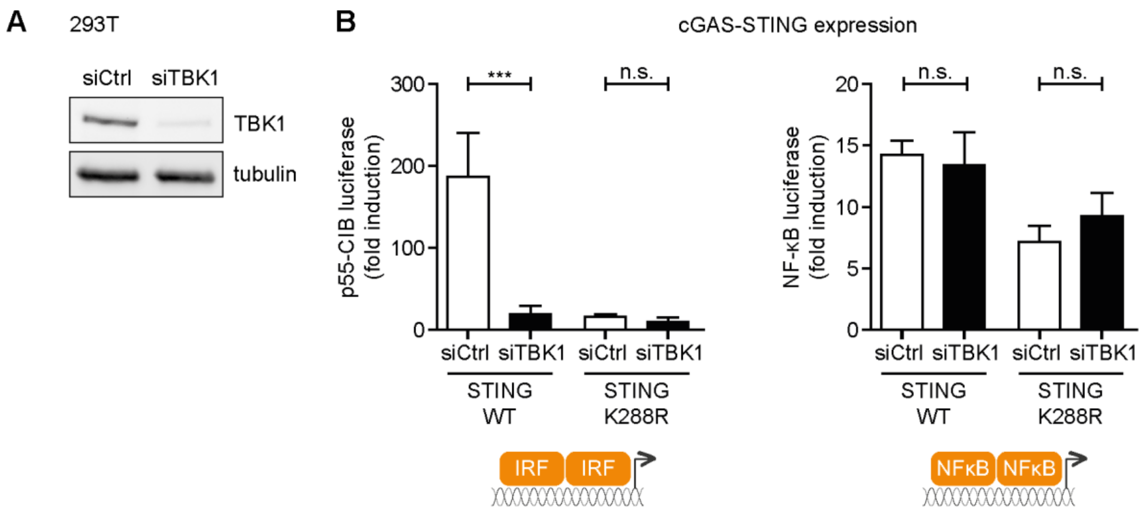

Appendix Figure S5: siRNA-mediated knockdown of TBK1 does not affect STING-dependent NF-κB activation (corresponding to Figure 8)

(A-B) (A) 293T cells reverse transfected with siRNA targeting TBK1 or corresponding control siRNA were analyzed by immunoblotting with antibodies specific for TBK1 and tubulin.

(B) 48 hours post siRNA transfection, 293T cells were co-transfected with Cherry-STING, pRL-TK, cGAS-GFP (stimulated) or IRES-GFP (unstimulated) and either the pNF-κB or p55-CIB luciferase reporter. 20 hours post transfection, cells were lysed and a dual-luciferase assay was performed. Data shown is representative of two independent experiments and shown as mean ± SD.

n.s. not significant, ***p<0.001
