# Supplementary material for: The herpesviral antagonist m152 reveals differential activation of STING‐dependent IRF and NF‐κB signaling and STING's dual role during MCMV infection
Source: EMBO J. 2019 Jan 29;38(5):e100983. doi: 10.15252/embj.2018100983 (PMC6396373; doi:10.15252/embj.2018100983)
Supplement: Supplementary file 2 — Expanded View Figures PDF [file EMBJ-38-e100983-s002.pdf]

# Expanded View Figures

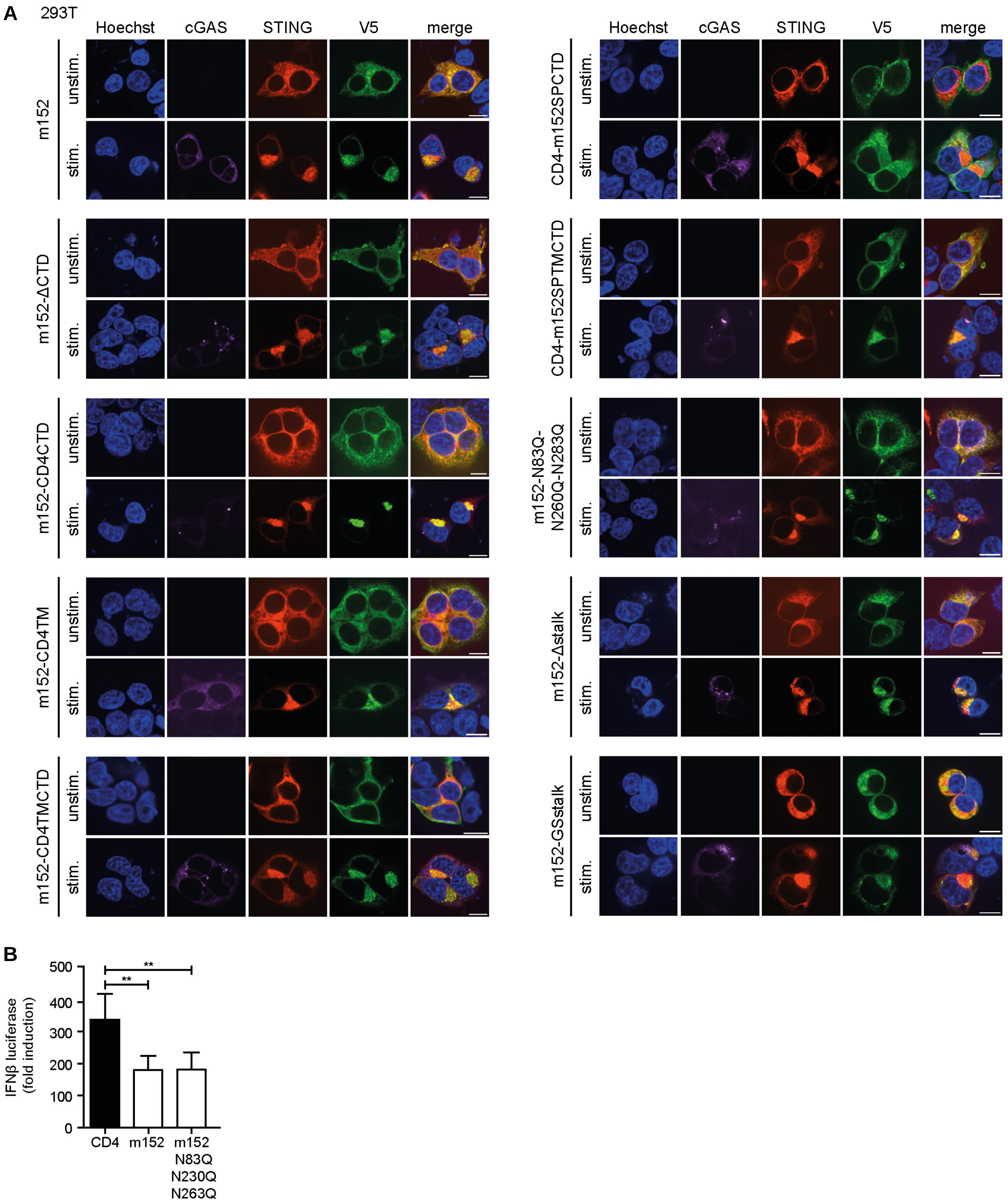


**Figure EV1.**

**Figure EV1. Subcellular localization of m152 mutant proteins in 293T cells.**

A 293T cells were co-transfected with expression plasmids for Cherry-STING, V5-tagged m152, or the respective m152 mutant (as described in Fig 3A) and either ev (unstimulated) or cGAS-GFP (stimulated). Twenty-four hours post-transfection, cells were fixed for immunolabeling with an anti-V5 antibody. Scale bar represents 10 μm.

B 293T cells were co-transfected with expression plasmids for Cherry-STING, IFNβ-Luc, pRL-TK and either CD4, m152 or the m152-N83Q-N230Q-N263Q mutant. For stimulation, samples were co-transfected with cGAS-GFP whereas unstimulated samples were co-transfected with IRES-GFP. A dual-luciferase assay was performed. Data are combined from three independent experiments and shown as mean ± SD.

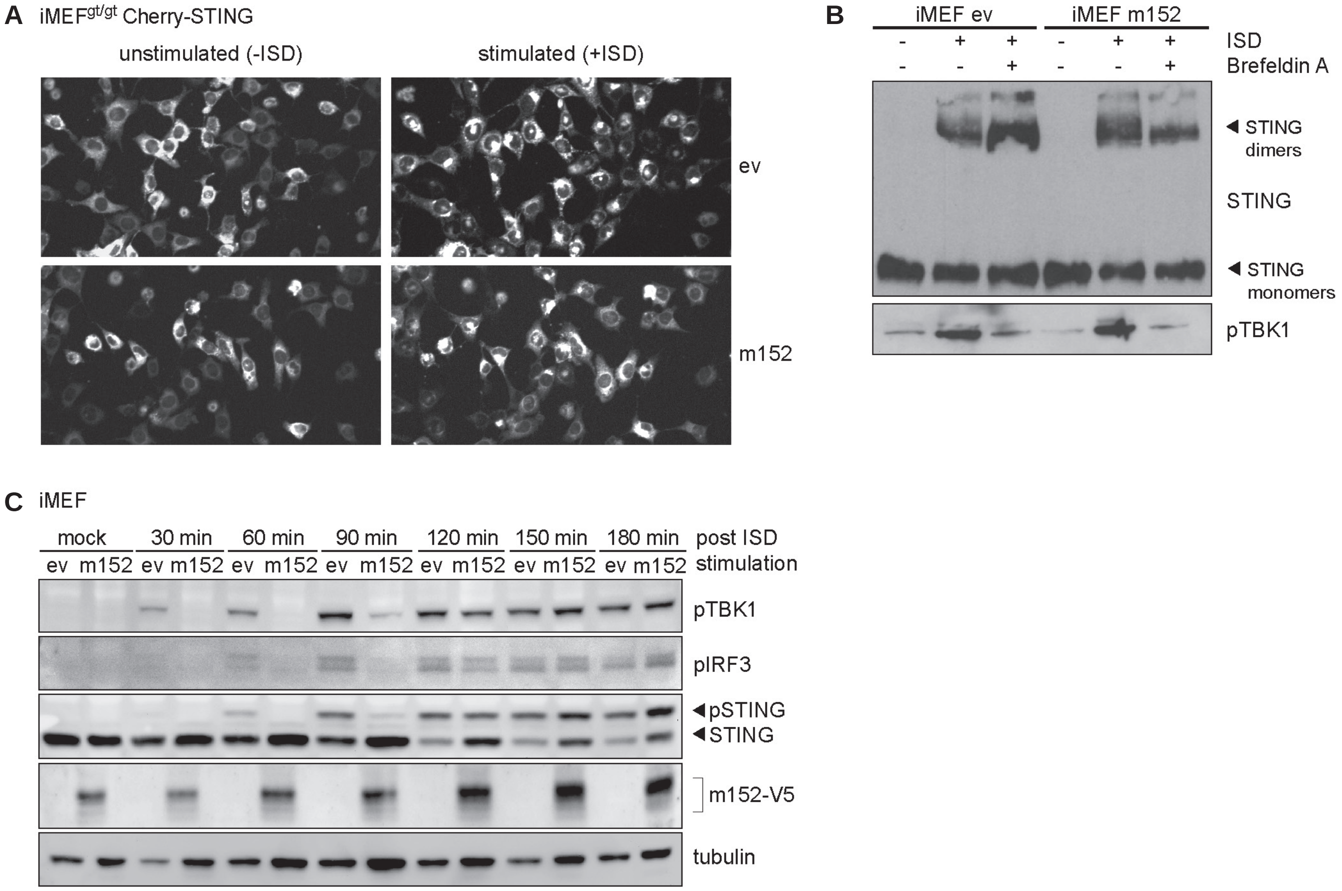


**Figure EV2. m152 delays the translocation of STING and downstream signaling.**

A Representative still images from live cell imaging experiments with iMEF$^{gt/gt}$ stably expressing Cherry-STING and either m152-V5 or empty vector (ev) transfected with ISD for 120 min (right panel) or left unstimulated (left panel).

B iMEF stably expressing m152-V5 and respective control cells (ev) were mock stimulated or stimulated with 10 μg/ml ISD in the presence or absence of Brefeldin A. 90 min post-stimulation, cell lysates were analyzed by SDS–PAGE under non-reducing conditions and subjected to immunoblotting with antibodies specific to STING and phospho-TBK1.

C iMEF stably expressing V5-tagged m152 or corresponding control cells (ev) were stimulated with 5 μg/ml ISD for the indicated time or left unstimulated (mock). Lysates were subjected to IB with the specified antibodies. IB shown are representative of two independent experiments.

Source data are available online for this figure.

 © 2019 The Authors

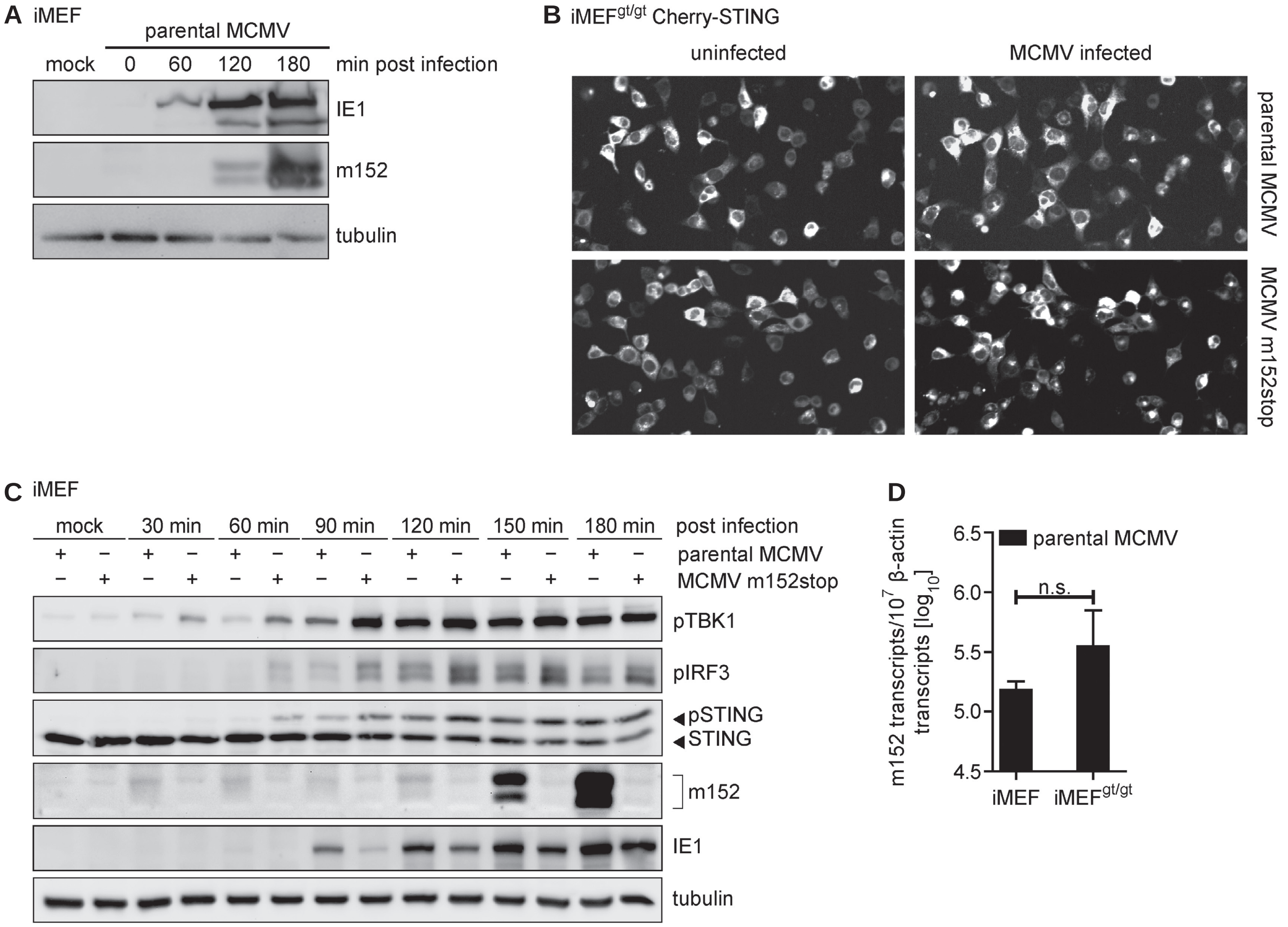


**Figure EV3. STING trafficking and downstream signaling is delayed in iMEF infected with parental MCMV compared to infection with MCMV m152stop.**

A iMEF were infected by centrifugal enhancement with parental MCMV at an MOI of 0.5 or mock infected. Cells were lysed at the indicated time points, and lysates were subjected to immunoblotting with antibodies specific to the MCMV proteins immediate-early protein 1 (IE1) and m152. Tubulin levels were determined with a tubulin antibody.

B Representative still images from live cell imaging experiments with iMEF$^{gt/gt}$ stably expressing Cherry-STING infected with the parental MCMV (upper panel) or MCMV m152stop (lower panel) at 120 min post-infection.

C iMEF were infected by centrifugal enhancement with parental MCMV or MCMV m152stop at an MOI of 0.1 or mock infected. Cells were lysed at the indicated time points, and lysates were subjected to immunoblotting with specified antibodies.

D iMEF or iMEF$^{gt/gt}$ were infected by centrifugal enhancement with parental MCMV at an MOI of 0.01. Six hpi, total RNA was extracted and *m152* transcript levels were determined by qRT–PCR. Data were normalized to $10^7$ cellular β-actin transcripts and are shown as mean ± SD.

Source data are available online for this figure.

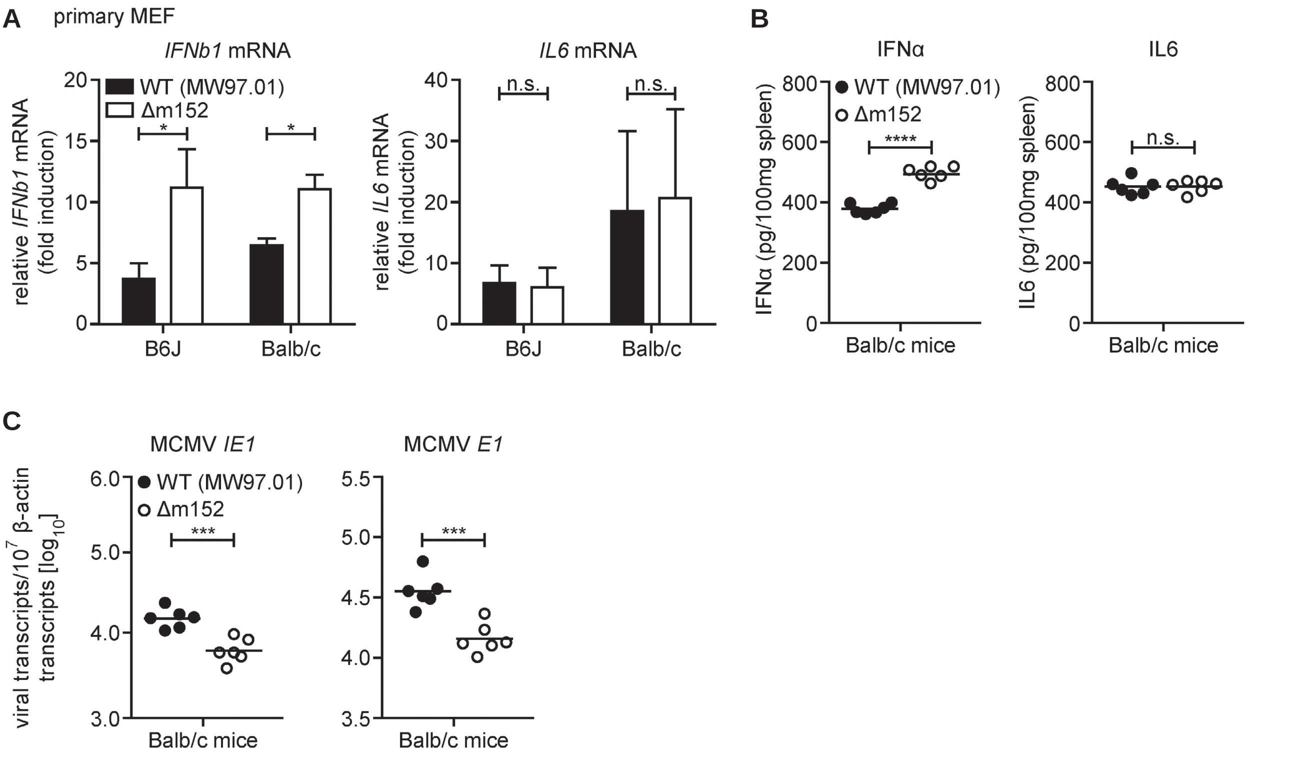


**Figure EV4. Modulatory effect of m152 on the type I IFN response is present in Balb/c mice.**

A Primary MEF from B6J or Balb/c mice were infected by centrifugal enhancement with MCMV WT (MW97.01) or MCMV Δm152 at an MOI of 0.1. Four hpi total RNA was extracted to determine *IFNb1* and *IL6* transcripts by qRT–PCR. Data were normalized to $10^7$ cellular β-actin transcripts and combined from three (left panel) or two (right panel) independent experiments.

B Balb/c mice were i.v. infected with $1 \times 10^6$ PFU MCMV WT (MW97.01) or MCMV Δm152. IFNα (left panel) and IL-6 (right panel) levels in spleen organ homogenates were analyzed 6 hpi by ELISA.

C Balb/c mice were i.v. infected with $1 \times 10^6$ PFU MCMV WT (MW97.01) or MCMV Δm152. Six hpi, RNA was extracted from spleen homogenates and MCMV *IE1* (left panel) and MCMV *E1* (right panel) transcript levels were determined by qRT–PCR. Data were normalized to $10^7$ cellular β-actin transcripts.

Data information: (B-C) $n = 6$ mice per group; n.s. not significant, *$P < 0.05$, ***$P < 0.001$, ****$P < 0.0001$.


© 2019 The Authors

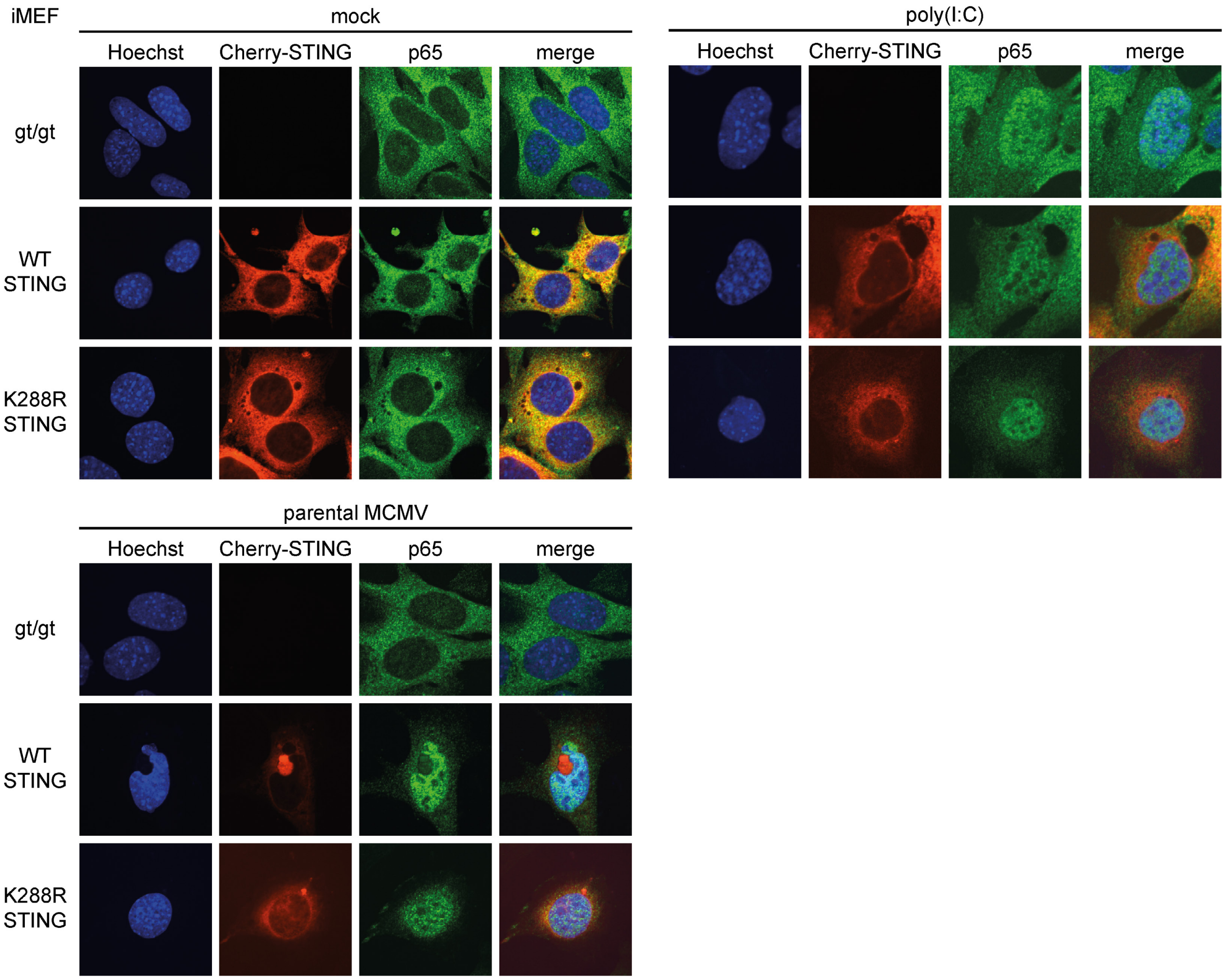


**Figure EV5. Nuclear translocation of p65 upon MCMV infection is dependent on STING.**

iMEF$^{gt/gt}$, iMEF$^{gt/gt}$ stably expressing Cherry-tagged WT STING, and iMEF$^{gt/gt}$ stably expressing Cherry-tagged K288R STING were mock treated, stimulated with 10 µg/ml poly (I:C) complexed with Lipofectamine, or infected by centrifugal enhancement with parental MCMV at an MOI of 0.05. Four hours post-stimulation or infection, cells were fixed for immunolabeling with an anti-p65 antibody. Scale bar represents 10 µm.
