# Supplementary figures and images for: The herpesviral antagonist m152 reveals differential activation of STING‐dependent IRF and NF‐κB signaling and STING's dual role during MCMV infection
Source: EMBO J. 2019 Jan 29;38(5):e100983. doi: 10.15252/embj.2018100983 (PMC6396373; doi:10.15252/embj.2018100983)

### Figure 2C

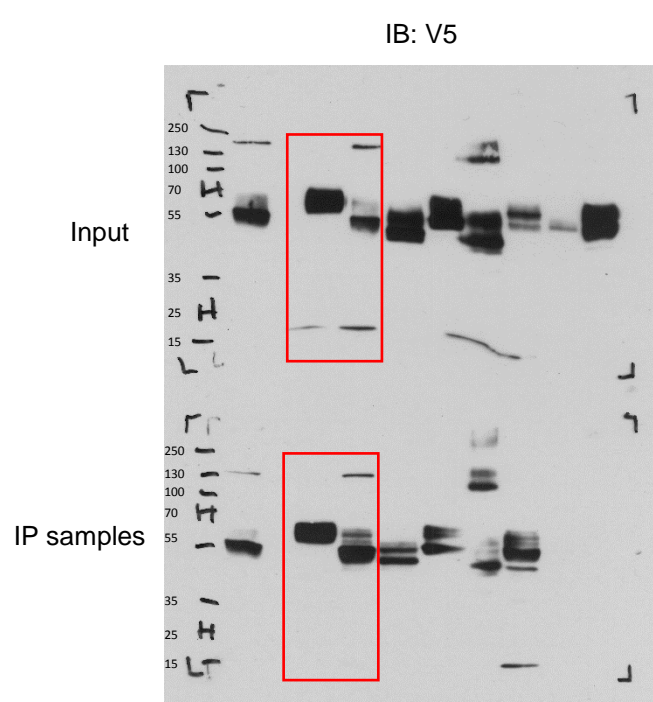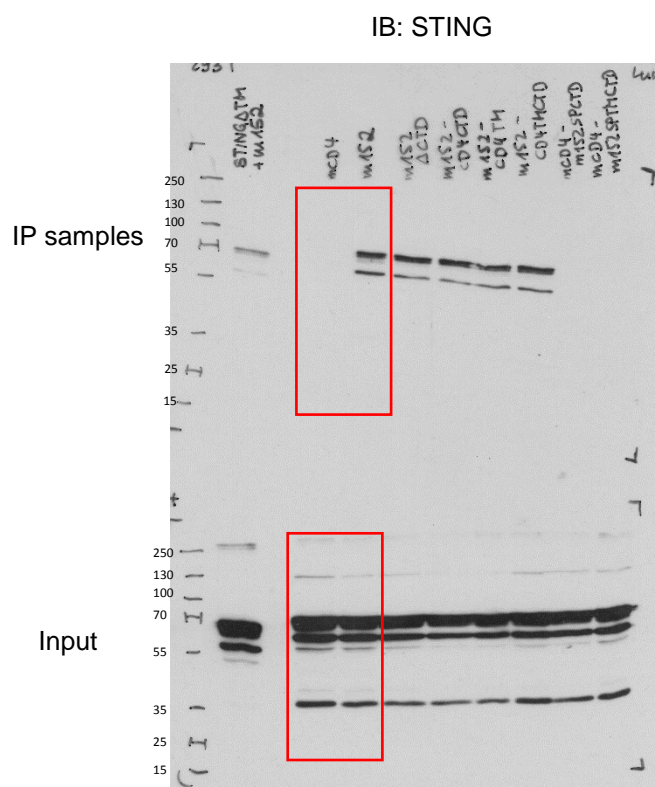

### Figure 2D

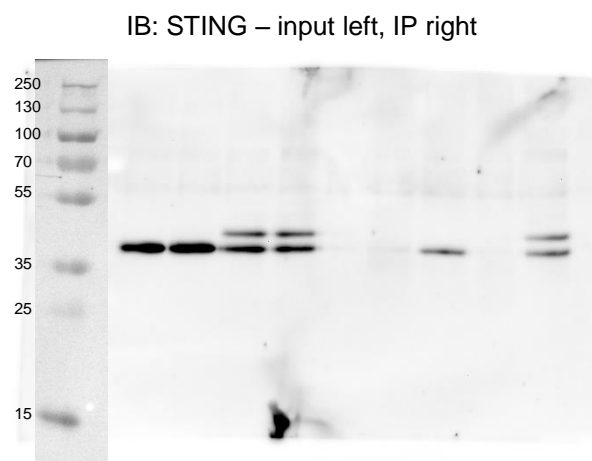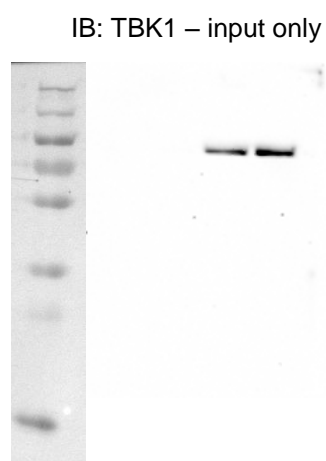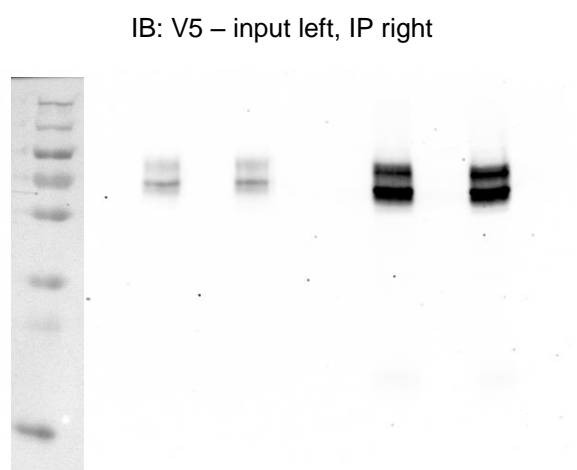

Supplement: Supplementary file 5 — Source Data for Figure 2 [file EMBJ-38-e100983-s003.pdf]

Source Data – Figure 3

Figure 3C

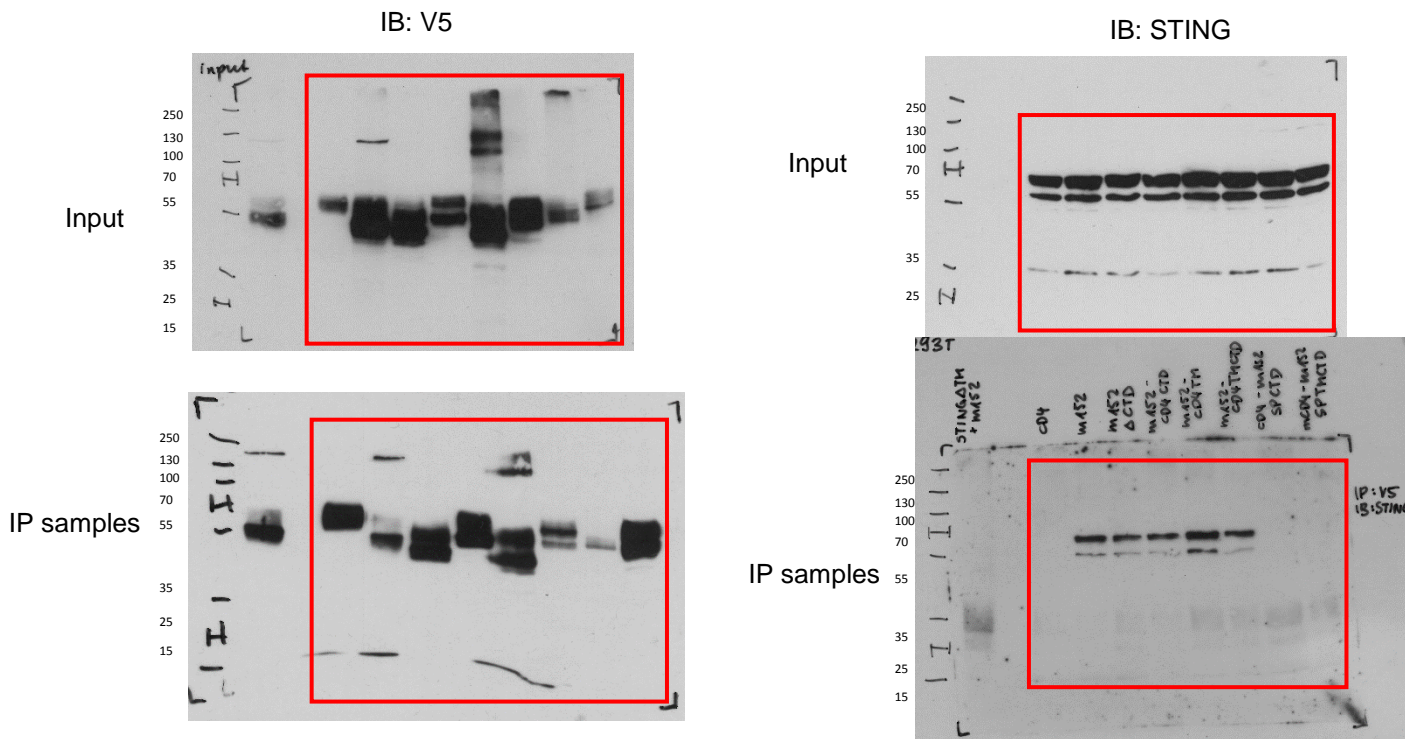

Figure 3D

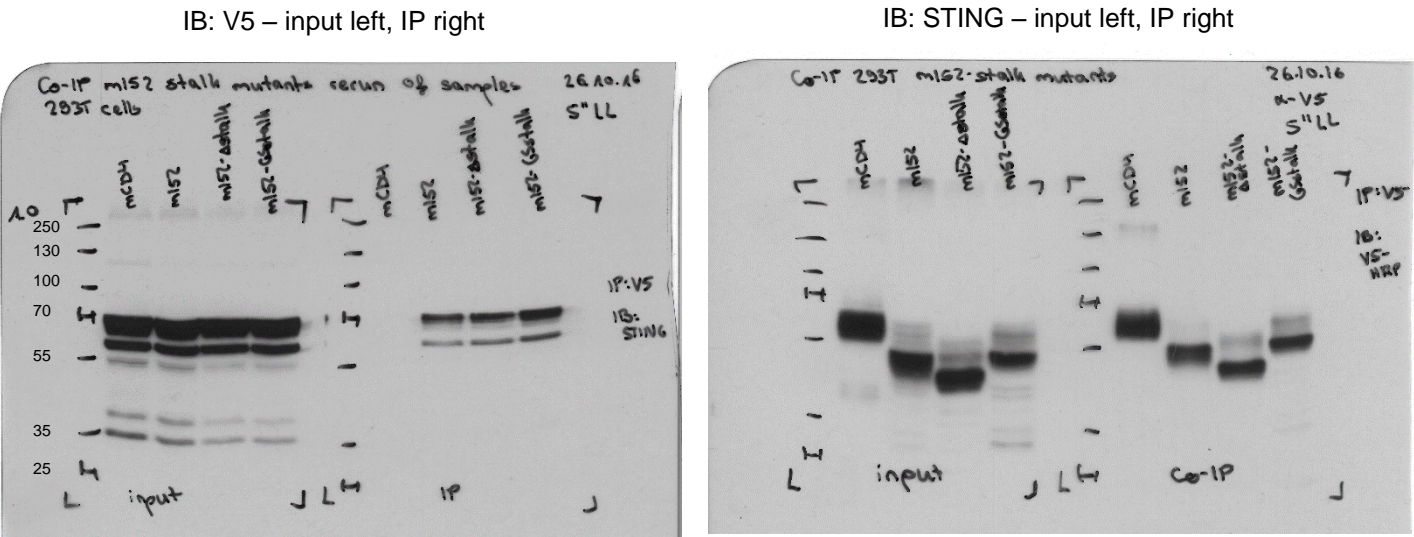

Supplement: Supplementary file 6 — Source Data for Figure 3 [file EMBJ-38-e100983-s004.pdf]

Source Data – Figure 4

Figure 4D

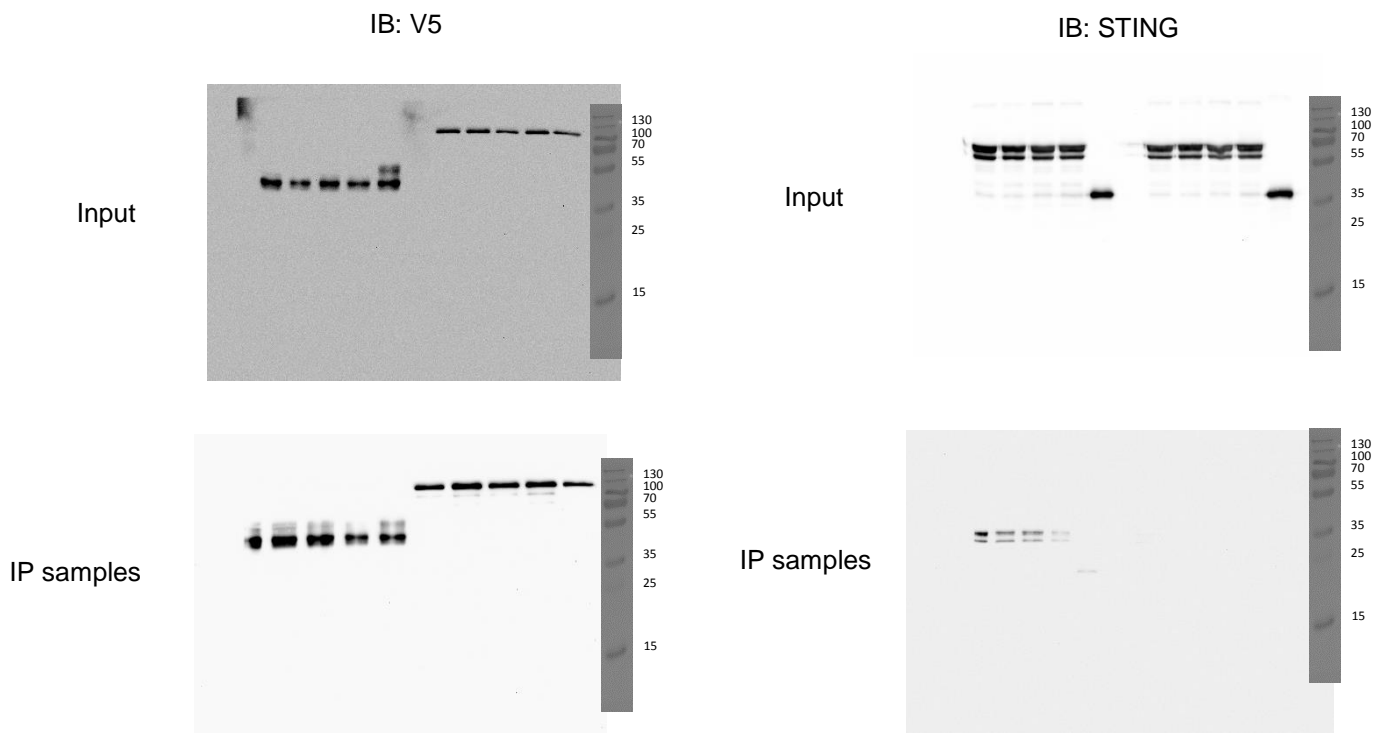

Figure 4F

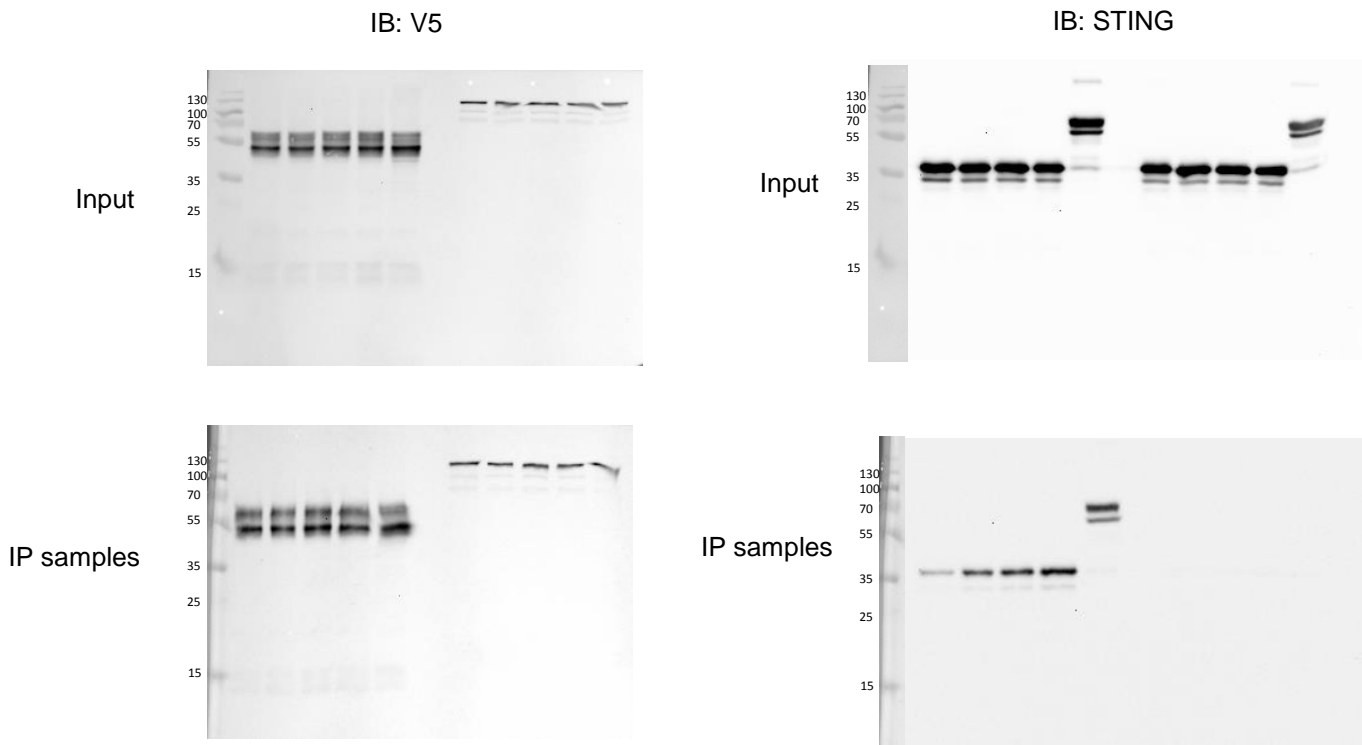

Supplement: Supplementary file 7 — Source Data for Figure 4 [file EMBJ-38-e100983-s005.pdf]

Source Data – Figure 5

Figure 5C

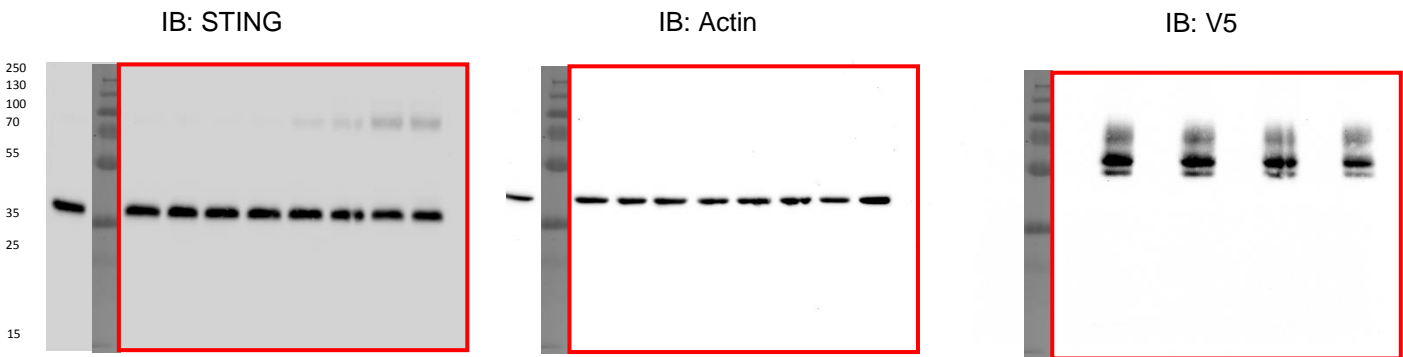

Figure 5F

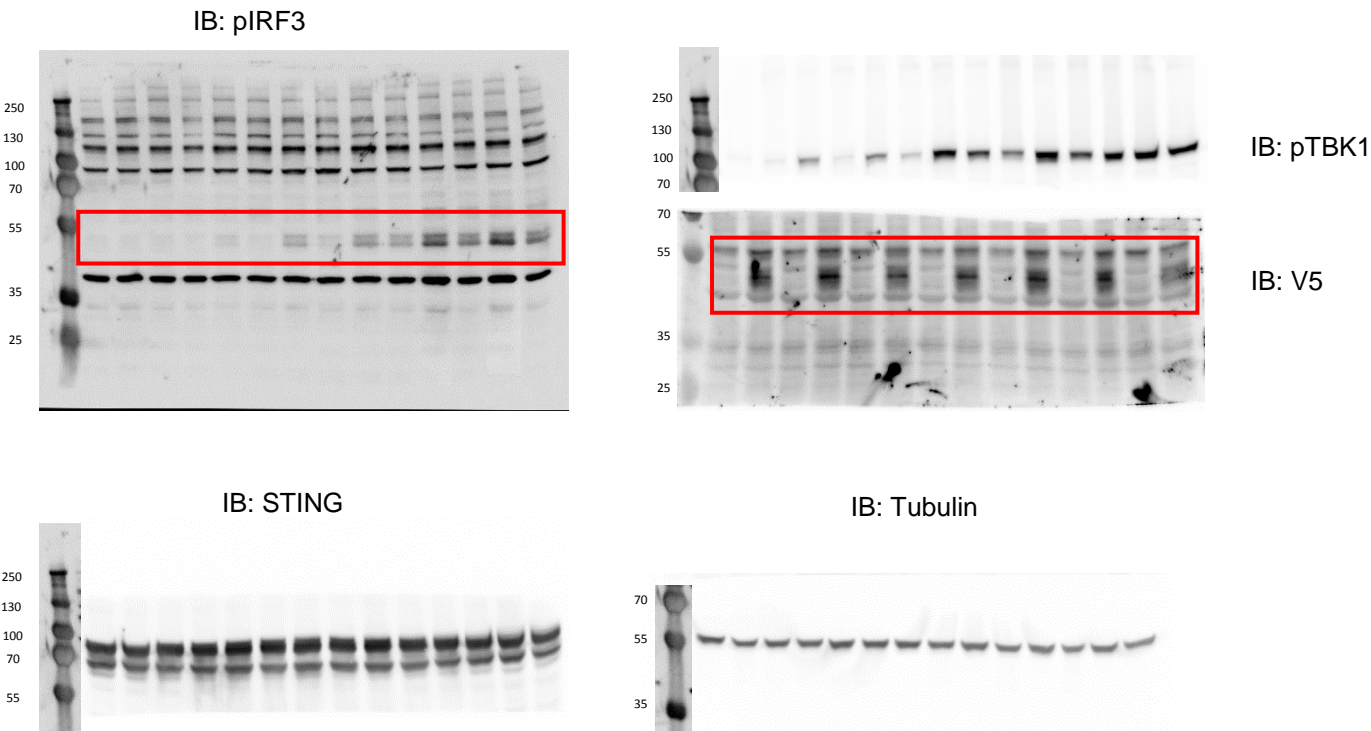

Supplement: Supplementary file 8 — Source Data for Figure 5 [file EMBJ-38-e100983-s006.pdf]

## Source Data – Figure 6

**Figure 6B**

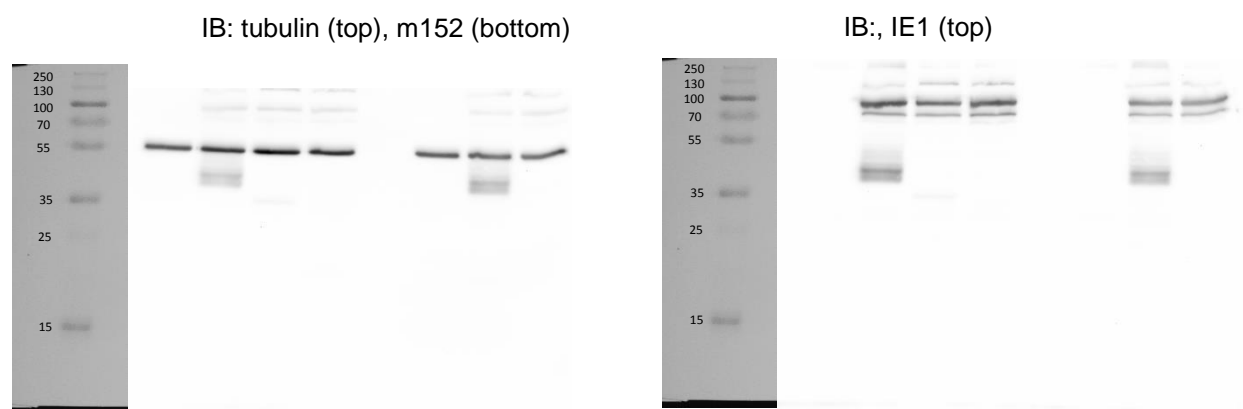

**Figure 6C**

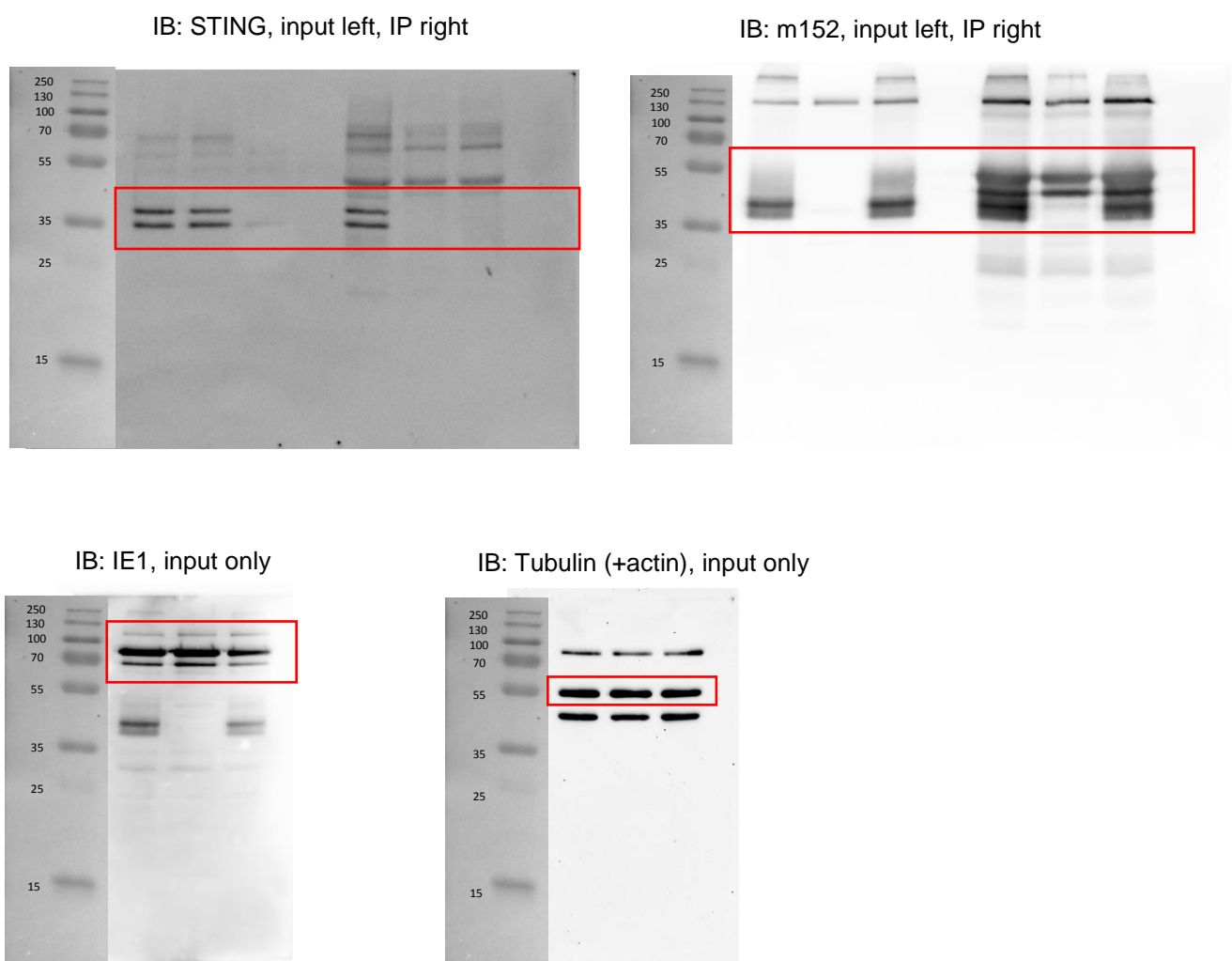

Supplement: Supplementary file 9 — Source Data for Figure 6 [file EMBJ-38-e100983-s007.pdf]
