# Supplementary material for: The herpesviral antagonist m152 reveals differential activation of STING‐dependent IRF and NF‐κB signaling and STING's dual role during MCMV infection
Source: EMBO J. 2019 Jan 29;38(5):e100983. doi: 10.15252/embj.2018100983 (PMC6396373; doi:10.15252/embj.2018100983)

Source Data – Appendix Figure S2

Appendix Figure S2A

IB: actin (top panel), V5 (middle panel), STING (lower panel)

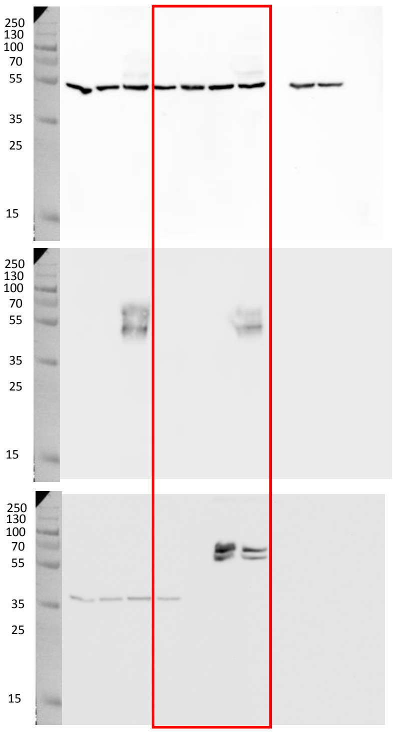

Appendix Figure S2C

IB: actin (top panel), V5 (middle panel), STING (lower panel)

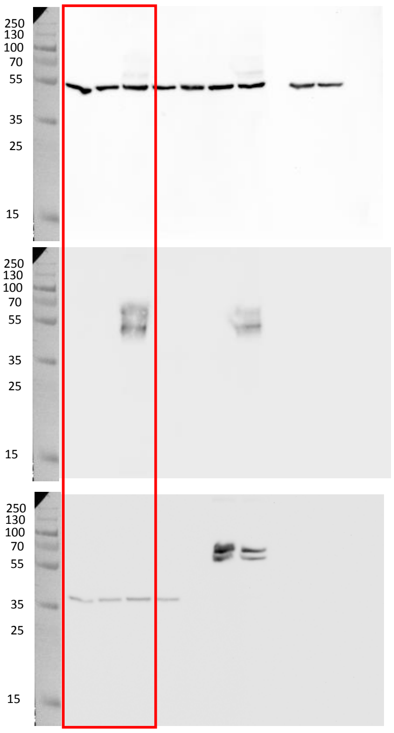

Appendix Figure S2B

IB: actin (left panel), V5 (right panel)

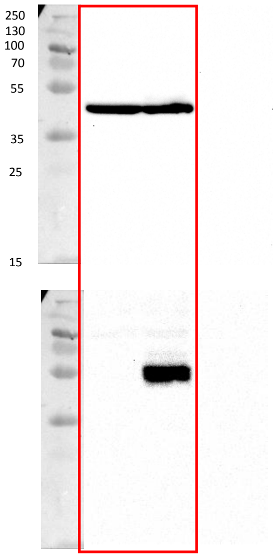

Supplement: Supplementary file 3 — Source Data for Expanded View and Appendix [file EMBJ-38-e100983-s008.zip › Source_Data_Appendix_Figure_S2A,B,C_and_S5_.zip!/92088_2_additional_figure_data_1398416_pjtn3p.pdf]
