# Supplementary material for: The herpesviral antagonist m152 reveals differential activation of STING‐dependent IRF and NF‐κB signaling and STING's dual role during MCMV infection
Source: EMBO J. 2019 Jan 29;38(5):e100983. doi: 10.15252/embj.2018100983 (PMC6396373; doi:10.15252/embj.2018100983)

Source Data – Figure EV2

Figure EV2B

IB: STING (monomers: lower bands, dimers: upper bands)

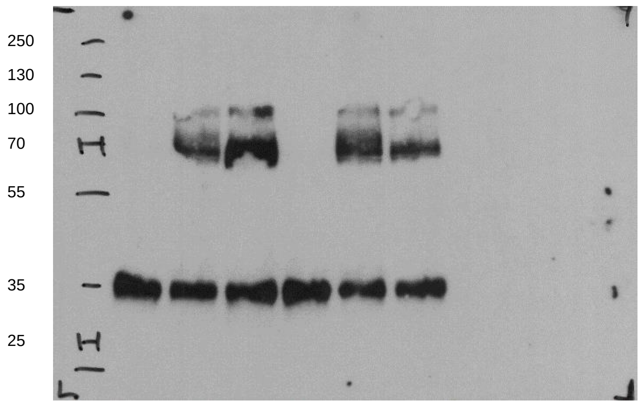

IB: pTBK1 (upper bands)

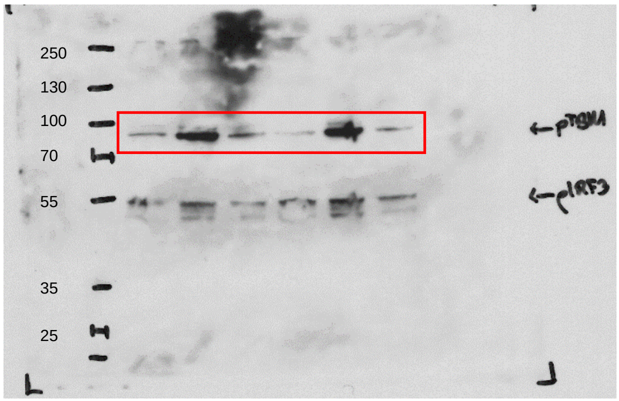

Figure EV2C

IB: STING (unphosphorylated: lower bands, phosphorylated: upper bands)

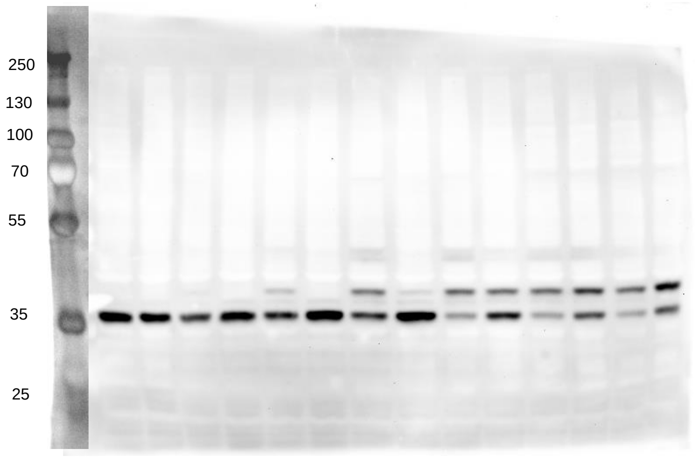

IB: pTBK1

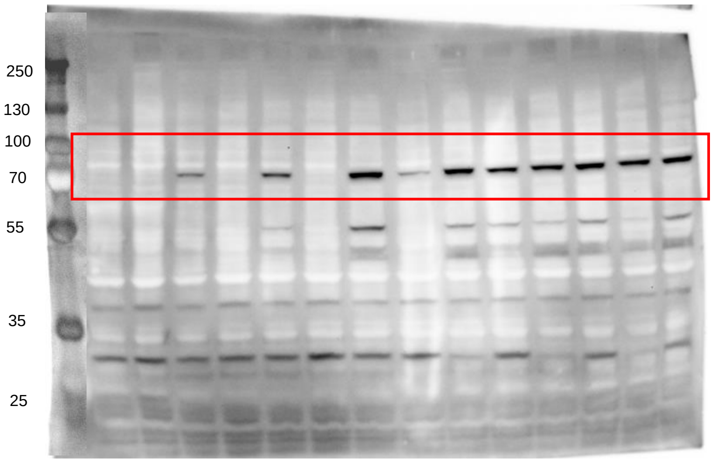

IB: V5

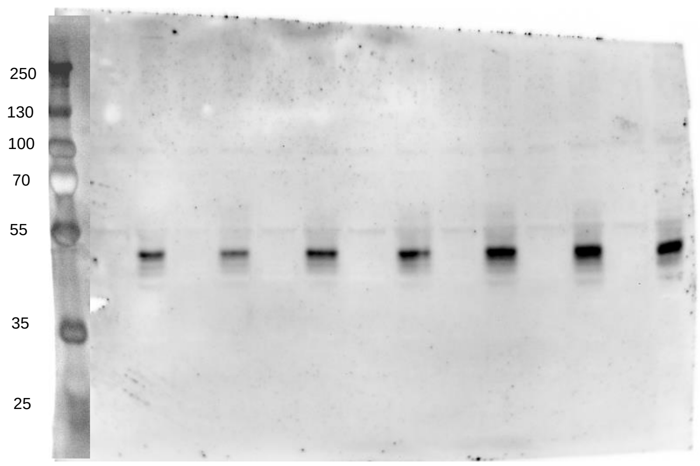

IB: pIRF3

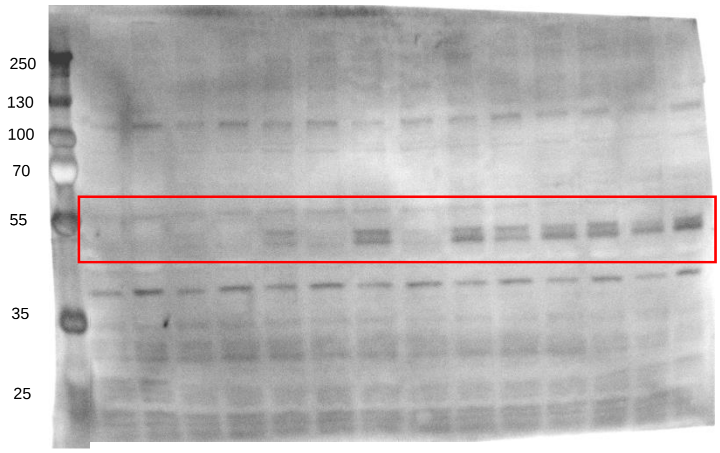

IB: tubulin

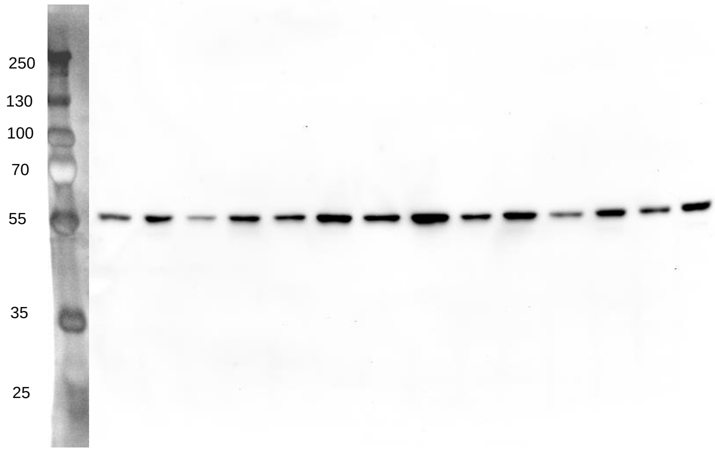

Supplement: Supplementary file 3 — Source Data for Expanded View and Appendix [file EMBJ-38-e100983-s008.zip › Source_Data_Fig_EV3_and_EV3.zip!/Source_Data_-_Figure_EV2.pdf]
